# Supplementary material for: Predicting visual function by interpreting a neuronal wiring diagram
Source: Nature. 2024 Oct 2;634(8032):113–23. doi: 10.1038/s41586-024-07953-5 (PMC11446822; doi:10.1038/s41586-024-07953-5)
Supplement: Supplementary file 6 — CRF and ERF predictions for individual TmY4 and TmY9 cells. Analogous to Supplementary Data 3, but for TmY target types. Shown are the top four monosynaptic pathways, the strongest pathway passing through each of the top ten intermediary types (ranking from Extended Data Fig. 7), and the trisynaptic pathway Tm1–TmY–Dm3–TmY (see the section entitled Prediction of spatial normalization). [file 41586_2024_7953_MOESM6_ESM.zip › DataS4/TmY9q/720575940634952184.pdf]

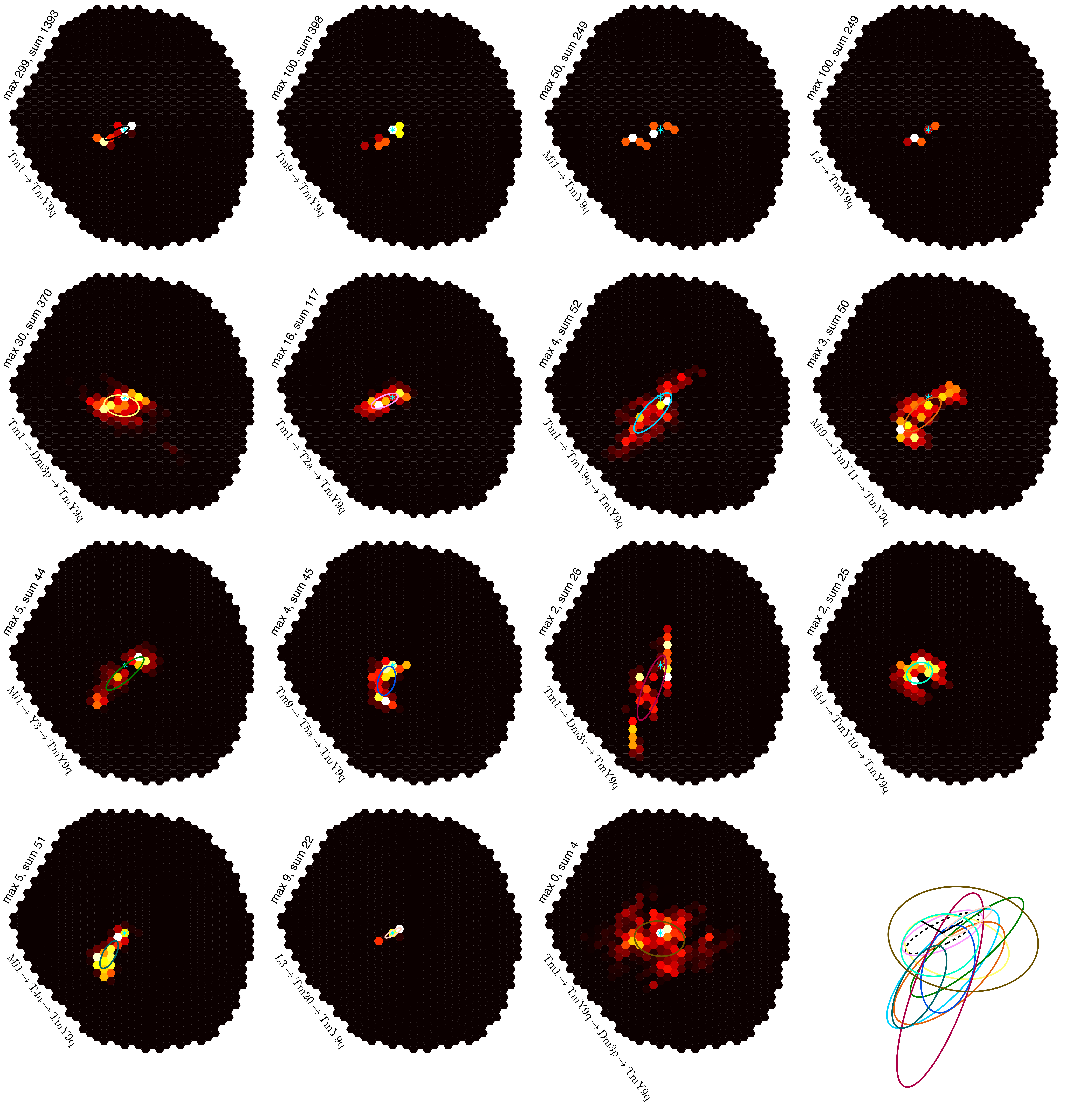
max 299, sum 1393
Tm1 → TmY9q
max 100, sum 398
Tm9 → TmY9q
max 50, sum 249
Mi1 → TmY9q
max 100, sum 249
L3 → TmY9q
max 30, sum 370
Tm1 → Dm3p → TmY9q
max 16, sum 117
Tm1 → T2a → TmY9q
max 4, sum 52
Tm1 → TmY9q → TmY9q
max 3, sum 50
Mi9 → TmY11 → TmY9q
max 5, sum 44
Mi1 → Y3 → TmY9q
max 4, sum 45
Tm9 → T5a → TmY9q
max 2, sum 26
Tm1 → Dm3v → TmY9q
max 2, sum 25
Mi4 → TmY10 → TmY9q
max 5, sum 51
Mi1 → T4a → TmY9q
max 9, sum 22
L3 → Tm20 → TmY9q
max 0, sum 4
Tm1 → TmY9q → Dm3p → TmY9q
